# Insecticide resistance status of *Aedes aegypti* in Bangladesh

**DOI:** 10.1186/s13071-020-04503-6

**Published:** 2020-12-14

**Authors:** Hasan Mohammad Al-Amin, Fatema Tuj Johora, Seth R. Irish, Muhammad Riadul Haque Hossainey, Lucrecia Vizcaino, Kishor Kumar Paul, Wasif A. Khan, Rashidul Haque, Mohammad Shafiul Alam, Audrey Lenhart

**Affiliations:** 1 Infectious Diseases Division, International Centre for Diarrhoeal Disease Research Bangladesh (icddr,b), 68 Shaheed Tajuddin Ahmed Sarani, Mohakhali, Dhaka, 1212 Bangladesh; 2 QIMR Berghofer Medical Research Institute (QIMR Berghofer), Brisbane, QLD 4006 Australia; 3 Division of Parasitic Diseases and Malaria, Center for Global Health, Centers for Disease Control and Prevention, Atlanta, GA USA; 4 President’s Malaria Initiative, Bureau for Global Health, Office of Infectious Disease, United Agency for International Development, Washington, DC USA; 5 Department of Biological Sciences, George Washington University, Washington, DC 20052 USA; 6 The Kirby Institute, University of New South Wales, Sydney, NSW Australia

**Keywords:** *Aedes aegypti*, Insecticide resistance, Bangladesh, Bioassays, Mortality, *Kdr*, Esterase, Oxidase

## Abstract

**Background:**

Arboviral diseases, including dengue and chikungunya, are major public health concerns in Bangladesh where there have been unprecedented levels of transmission reported in recent years. The primary approach to control these diseases is to control the vector *Aedes aegypti* using pyrethroid insecticides. Although chemical control has long been practiced, no comprehensive analysis of *Ae. aegypti* susceptibility to insecticides has been conducted to date. The aim of this study was to determine the insecticide resistance status of *Ae. aegypti* in Bangladesh and investigate the role of detoxification enzymes and altered target site sensitivity as resistance mechanisms.

**Methods:**

Eggs of *Aedes* mosquitoes were collected using ovitraps from five districts across Bangladesh and in eight neighborhoods of the capital city Dhaka, from August to November 2017. CDC bottle bioassays were conducted for permethrin, deltamethrin, malathion, and bendiocarb using 3- to 5-day-old F_0_–F_2_ non-blood-fed female mosquitoes. Biochemical assays were conducted to detect metabolic resistance mechanisms, and real-time PCR was performed to determine the frequencies of the knockdown resistance (*kdr*) mutations Gly1016, Cys1534, and Leu410.

**Results:**

High levels of resistance to permethrin were detected in all *Ae. aegypti* populations, with mortality ranging from 0 to 14.8% at the diagnostic dose. Substantial resistance continued to be detected against higher (2×) doses of permethrin (5.1–44.4% mortality). Susceptibility to deltamethrin and malathion varied between populations while complete susceptibility to bendiocarb was observed in all populations. Significantly higher levels of esterase and oxidase activity were detected in most of the test populations as compared to the susceptible reference Rockefeller strain. A significant association was detected between permethrin resistance and the presence of Gly1016 and Cys1534 homozygotes. The frequency of *kdr* (knockdown resistance) alleles varied across the Dhaka *Aedes* populations. Leu410 was not detected in any of the tested populations.

**Conclusions:**

The detection of widespread pyrethroid resistance and multiple resistance mechanisms highlights the urgency for implementing alternate *Ae. aegypti* control strategies. In addition, implementing routine monitoring of insecticide resistance in *Ae. aegypti* in Bangladesh will lead to a greater understanding of susceptibility trends over space and time, thereby enabling the development of improved control strategies.
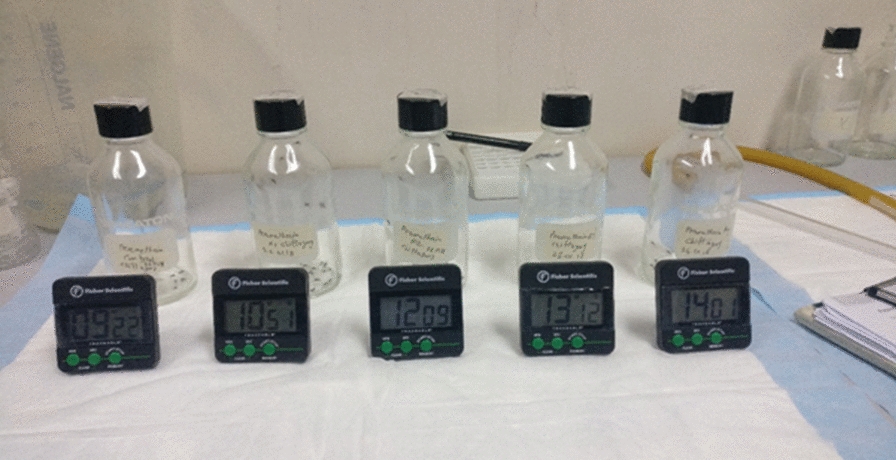

## Background

*Aedes* (*Stegomyia*) *aegypti* (Linnaeus, 1762) is an important vector of arboviral diseases, principally dengue, chikungunya, and Zika. These increasingly common arboviral infections cause severe febrile illness and short- to long-term physical or cognitive impairments and even death. Dengue is the most prevalent and rapidly spreading arboviral disease worldwide, with an estimated 390 million annual infections and 3.9 billion people at risk [1]. Chikungunya is also increasingly prevalent, and the prolonged pain and rheumatism resulting from infection can result in long-term physical problems and impaired daily life [2, 3]. Zika recently caused a major global pandemic in 2015–2016, leading to congenital malformations, Guillain–Barre syndrome, and other severe neurological complications [4].

The burden of arboviral diseases in Bangladesh is not well documented. The first major outbreak of dengue took place during the monsoon of 2000 and caused 5521 officially reported cases with 93 deaths [5]. Since then, thousands of infections are reported each year although these numbers represent a fraction of the actual burden since only patients admitted to some selected hospitals are officially reported [6]. Recent estimates suggest that 40 million people have been infected nationally, with an average of 2.4 million infections annually. Cases are mostly concentrated in the capital city Dhaka, where the seropositivity ranges from 36 to 85% [7]. In 2019, Bangladesh experienced its largest outbreak, with 101,354 confirmed cases and 164 deaths [8]. Since 2008, sporadic infections with chikungunya virus have been reported across Bangladesh, with the largest outbreak occurring in 2017 during which hundreds of thousands of inhabitants of Dhaka were infected [9]. Zika virus transmission has not been widely reported in Bangladesh, with only a single confirmed case in 2016 in a 67-year old man from Chittagong who had not traveled outside of Bangladesh. Although a few additional Zika virus infections have been detected by antibody tests, there is no further evidence of Zika in Bangladesh [10, 11].

*Aedes aegypti* is highly abundant throughout Bangladesh, especially in Dhaka [7]. In 2018, the Breteau index (BI; the number of *Aedes*-positive containers per 100 houses inspected) was > 100 in some parts of Dhaka [12]. Recent studies in Dhaka have confirmed that plastic containers (plastic drums, buckets, plastic bags, bottles, and disposable cups) and discarded vehicle and construction materials (tires, battery shells, and cement mixers) are key containers for *Aedes* production. These are typical of the domestic and industrial detritus that facilitate the proliferation of *Ae. aegypti* across the globe. High *Aedes* abundance in Dhaka is also strongly associated with favorable climatic factors, including rainfall, temperature, and humidity [13].

In the absence of effective therapeutic drugs and vaccines, *Ae. aegypti* control is presently the only approach for preventing and controlling the transmission of most *Aedes*-borne arboviruses. *Aedes aegypti* control strategies rely heavily on the application of a limited number of chemical insecticides approved for public health use, principally pyrethroids, organochlorines, organophosphates, and carbamates [14]. Of these, pyrethroid insecticides, such as deltamethrin, cypermethrin, and permethrin, are commonly used because of their low toxicity to mammals and their high efficacy against vectors. However, resistance to many insecticides has emerged in *Ae. aegypti* across the globe, representing a serious threat to control programs [15–19].

Resistance to insecticides is a dynamic evolutionary process that is driven by insecticide selection pressures [20]. Resistance can be caused by physiological changes, including (i) changes to the mosquito cuticle so insecticides cannot penetrate, (ii) increased activity of insecticide detoxification enzymes, and/or (iii) structural modifications at the target site of the insecticide; it can also result from behavioral adaptations, such as insecticide avoidance [21].

Alterations in the target sites as a result of resistance to pyrethroids and dichlorodiphenyltrichloroethane (DDT) are often caused by mutations in the voltage-gated sodium channel (VGSC) transmembrane protein and are broadly referred to as ‘knockdown resistance’ (*kdr*) mutations. There are several point mutations on the VGSC gene known to confer *kdr*-type insecticide resistance in *Ae. aegypti*, most notably at positions 410, 989, 1016, and 1534 [18, 22, 23]. Increased enzyme activity resulting in metabolic resistance typically involves any of the three main groups of detoxification enzymes: carboxylesterases, mixed-function oxidases (MFOs), and glutathione *S*-transferases (GSTs) [24]. Understanding the mechanisms of resistance and their specificity among insecticides is important to devising strategies to mitigate and manage insecticide resistance when it is detected.

Although there has been a recognized increase in *Aedes*-borne arboviruses in Bangladesh over the last 20 years, little or no organized use of insecticides against *Ae. aegypti* has occurred. Regular control activities are mostly carried out only in Dhaka, targeting the nuisance biting mosquitoes *Culex quinquefasciatus* and *Aedes* by thermal fogging with a combination of pyrethroid insecticides, including permethrin, prallethrin, and tetramethrin/bioallethrin. The increasing number of reported cases of *Aedes*-borne viral diseases indicate that the insecticides being used are having little impact. Development of resistance against commonly used insecticides in local *Aedes* populations may contribute to the failure of the vector control strategy. Occasional source reduction is also carried out by community engagement by both government and private initiatives. However, gaining access to all premises and achieving sufficient coverage of myriad oviposition sites in densely populated cities like Dhaka is a huge challenge [25]. There are also structural challenges to control activities related to management, evaluation, and budget [26, 27].

The insecticide resistance status of *Ae. aegypti* has not previously been comprehensively assessed in Bangladesh. The purpose of this study was to assess the insecticide resistance status and resistance mechanisms of key *Ae. aegypti* populations in Bangladesh to better inform future insecticide choices for vector control.

## Methods

### Study sites

**Fig. 1:**
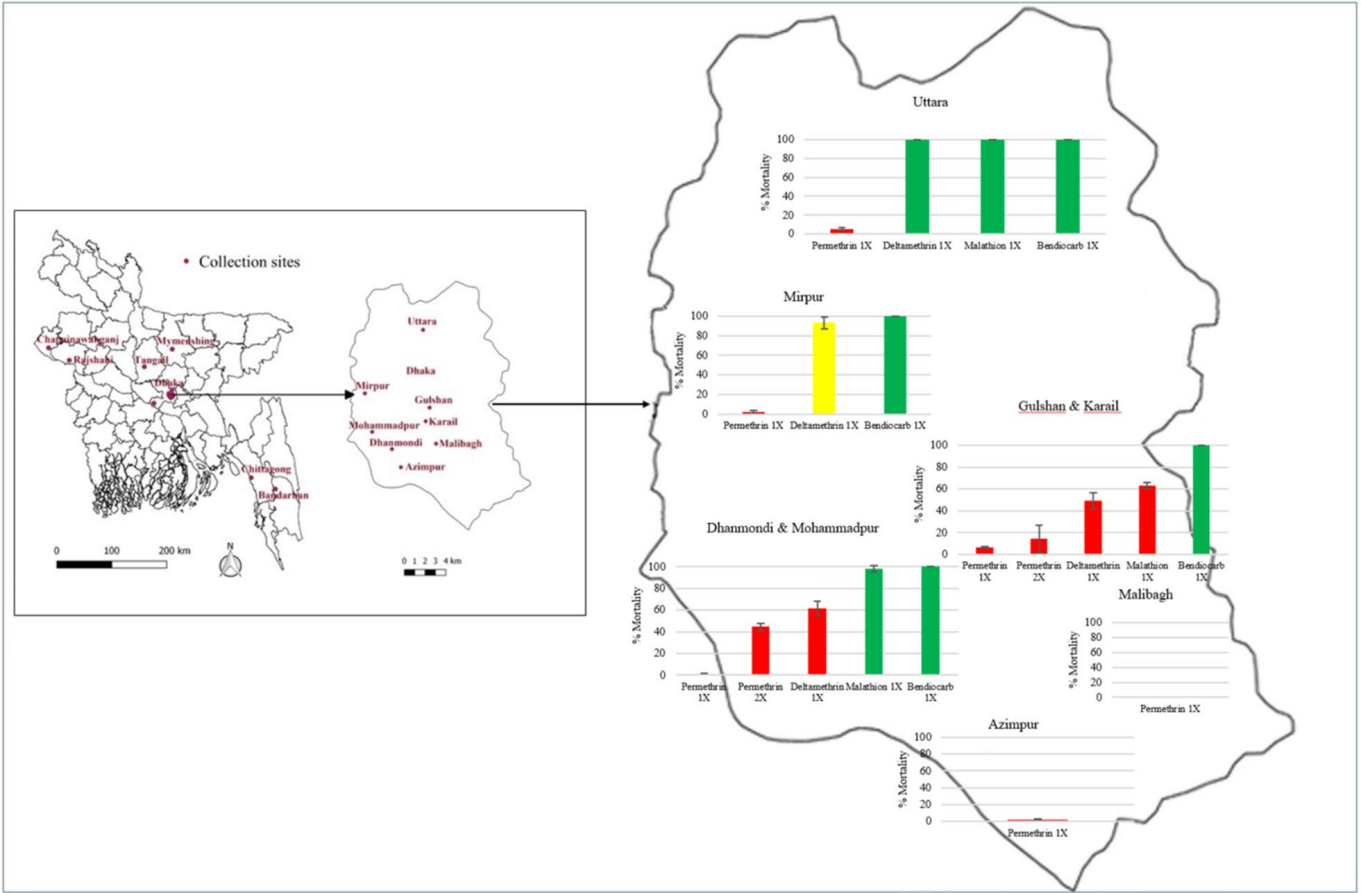
Bioassay results for female *Aedes aegypti* in eight areas in Dhaka City. Red bars indicate resistance, green bars indicate susceptibilit,y and yellow bars indicate developing resistance. Results are given as percentage mortality at 30 min ± 95% confidence interval (CI)

Mosquitoes were collected from five districts throughout Bangladesh. Of these, the capital city, Dhaka, and the port city, Chittagong are high-transmission settings and the city of Rajshahi and district of Chapai Nawabganj are low-transmission settings [9, 28–32] (Fig. 1). The other district, Bandarban, was selected as it is a malaria-endemic region, and as preventative measures deltamethrin-impregnated long-lasting insecticidal nets (LLINs) are regularly distributed among the population and there is seasonal sporadic indoor residual spraying (IRS) [33, 34]. Since the majority of *Aedes*-borne arboviral infections are reported from Dhaka, eight areas within Dhaka City were selected for sampling [35].

### Collection of *Aedes* eggs

Eggs were collected using ovitraps baited with a grass infusion. Ovitraps consisted of black 2-l containers made of plastic and an oviposition substrate of brown seed germination paper. The ovitraps were filled with 50 ml of 2- to 3-day-old grass infusion and 1200 ml of tap or rainwater. After verbal consent was obtained from household owners, the ovitraps were placed primarily indoors, including the main living area (under beds), behind refrigerators, under stairways, in garages, and on balconies. When these sites were not suitable, ovitraps were set in the yards under sheds close to the house. Within Dhaka City, the number of ovitraps varied from 50 to 70 per location, whereas in the areas outside of Dhaka (non-Dhaka), approximately 100 ovitraps were set in each location. For all non-Dhaka districts except Chittagong, eggs were collected from one urban and one rural location. All eggs were collected in 2017 during the traditional peak dengue transmission months of August to November.

### Mosquito rearing

Ovitraps were collected after 6 days* in situ*. Upon collection, the germination papers were dried and sent to the insectary at the Animal Research Facilities, International Centre for Diarrhoeal Disease Research, Bangladesh (icddr,b) in Dhaka. Due to unexpectedly long time required to prepare the rearing facility, mosquito rearing was delayed until December 2017. Given this delay, hatching rates were low for several locations, and eggs from adjacent locations were sometimes merged into a single population (Table 1). Mosquitoes were reared at a constant temperature (26–28 °C) and humidity (70–80%). When possible, mosquitoes were reared to the F2 generation to obtain sufficient numbers for a wide range of susceptibility tests. Artificial blood-feeding was provided using the methods described by Costa-da-Silva et al. [36]. Adult mosquitoes were provided with 10% sucrose solution. In addition to the field populations, the ‘Rockefeller’ (ROCK) *Ae. aegypti* reference strain, which is insecticide susceptible, was obtained from the U.S. Centers for Disease Control and Prevention (CDC, Atlanta, GA, USA) and reared as a susceptible control in the bioassays.

**Table 1:** Summary of *Aedis aegypti* populations tested in the study

Ovitrap collection sites			Final population^a^ for bioassay	Generation tested
General location	District	Specific location		
Dhaka	Dhaka	Azimpur	Azimpur	F0
		Dhanmondi	Dhahmondi & Mohammadpur	F0–F2
		Mohammadpur		
		Gulshan	Gulshan & Karail	F0–F2
		Karail		
		Mipur	Mirpur	F1–F2
		Malibagh	Malibagh	F2
		Uttara	Uttara	F1–F2
Non-Dhaka	Rajshahi	Rajshahi City (urban)	Rajshahi	F2
		Poba (rural)		
	Chapai Nawabganj	Chapai Nawabganj City (urban)	Chapai Nawabganj	F2
		Shibganj (rural)		
	Bandarban	Bandarban City (urban)	Bandarban	F0–F2
		Rowangchhari (rural)	No *Ae. aegypt;* all were *Ae. albopictus*	NA
	Chittagong	Chittagong City	Chittagong	F0–F2

^a^For ease of description, mosquitoes from each location are considered as a single population. Due to low hatching rates for some locations, eggs from adjacent locations were sometimes merged into a single population

### Insecticide susceptibility testing

Susceptibility tests were conducted following the CDC bottle bioassay protocol [37] using 3- to 5-day-old, non-blood-fed female mosquitoes. Four insecticides belonging to three major chemical classes were tested for each population when sufficient mosquitoes were available: (i) the pyrethroids permethrin and deltamethrin; (ii) the organophosphate malathion; (iii) the carbamate bendiocarb. Mosquitoes were exposed to diagnostic dose of each insecticide, which was 15 µg/bottle for permethrin, 10 µg/bottle for deltamethrin, 50 µg/bottle for malathion, and 12.5 μg/bottle for bendiocarb. When resistance was detected and sufficient mosquitoes were available, resistance intensity assays were also conducted by exposing mosquitoes to insecticide levels that were two- and fivefold (2× and 5×, respectively) higher than the respective diagnostic dose. All bioassays comprised > 100 mosquitoes per insecticide per population across four test bottles and 15–25 mosquitoes in an untreated control bottle. Susceptibility status was recorded after 0, 15, and 30 min of insecticide exposure; at each time point, mosquitoes unable to stand were considered to be dead [37]. Mortality data were interpreted according to World Health Organization (WHO) recommendations, with < 90% mortality in a population corresponding to resistance [38].

### Biochemical assays

Biochemical assays were performed to detect potential metabolic mechanisms of resistance through the altered activity of detoxifying enzymes. From each population, thirty 1- to 2-day-old female mosquitoes were tested for non-specific β-esterases (β-EST), MFOs, acetylcholine esterase (AChE), and insensitive acetylcholine esterase (IAChE), with a protein assay conducted for each mosquito to control for differences in body size. All mosquitoes were freeze killed and kept at − 20 °C until analysis. Briefly, mosquitoes were individually homogenized in 100 µl of potassium phosphate buffer followed by dilution to 2 ml with additional buffer. For all tests, mosquito homogenates were run in triplicate on 96-well round-bottom microplates (Corning Inc., Corning, NY, USA). Homogenates of the ROCK insecticide-susceptible *Ae. aegypti* reference strain were used as a comparator.

For the β-EST assay, 100 µl of mosquito homogenate was added to each well followed by 100 µl β-naphthyl acetate. The plate was then incubated at room temperature for 20 min, following which 100 µl Fast Blue was added in each well and the plate was further incubated at room temperature for 4 min at which time absorbance was read on a spectrophotometer (BioTek Instruments Inc., Winooski, VT, USA) using a 540-nm filter.

For the MFO assay, 100 µl of mosquito homogenate was added to each well followed by 200 µl of 3,3,5,5-tetramethylbenzidine (TMBZ) and 25 µl 3% hydrogen peroxide. The plate was incubated for 10 min and absorbance was read on a spectrophotometer using a 620-nm filter.

For the AChE assay, 100 µl of mosquito homogenate was added to each well followed by 100 µl of acetylthiocholine iodide (ATCH) and 100 µl dithio-bis-2-nitrobenzoic acid (DTNB). Absorbance was read immediately (*T*_0_) using a 414-nm filter, and a second reading was taken after 10 min (*T*_10_). The absorbance at *T*_0_ was subtracted from *T*_10_ and used as the value for data analysis.

The IAChE assay was similar to the AChE assay, with the addition of propoxur to the ATCH to quantify the extent to which propoxur inhibited the reaction.

The total protein content of each mosquito was measured by adding 20 µl of the homogenate to a well together with 80 µl of potassium phosphate and 200 µl of protein dye. Absorbance was read immediately using a 620-nm filter. Due to unforeseen circumstances, we were unable to run a standard curve with the same reagents employed in the total protein assay of the mosquitoes, precluding precise quantification of protein content.

### DNA extraction

DNA extraction was carried out using the REDExtract-N-Amp™ tissue kit (Merck KGaA, Darmstadt, Germany) according to the manufacturer’s protocol. Briefly, individual female mosquitoes were placed in 1.5-ml microcentrifuge tubes and mixed with 100 µl of extraction solution and 25 µl of tissue preparation solution. The tubes were then incubated at room temperature for 10 min followed by further incubation for 3 min at 95 °C. Then, 100 µl of neutralization solution B was added to the sample and the sample mixed by vortexing.

### Detection of *kdr* alleles (Gly1016, Cys1534, and Leu410)

To understand the correlation between phenotypic resistance and the presence of the *kdr* alleles Gly1016, Cys1534C, and Leu410, phenotyped mosquitoes exposed to permethrin and deltamethrin in the bioassays were assayed by real-time PCR. An additional 30 non-phenotyped mosquitoes from each of the six Dhaka populations were analyzed to estimate allele frequencies at the population level.

The Gly1016 PCR was performed following the protocol described by Saavedra-Rodriguez et al. [39]. Each reaction contained 4.5 µl of iQ-SYBR Green Supermix (Bio-Rad Laboratories Inc., Hercules, CA, USA), 0.45 µl of each primer, one common Gly forward primer (5ʹ-ACC GAC AAA TTG TTT CCC-3ʹ), one reverse primer for either Val (5ʹ-GCG GGC AGC AAG GCT AAG AAA AGG TTA ATT A-3ʹ) or Gly (5ʹ-GCG GGC AGG GCG GGG GCG GGG CCA GCA AGG CTA AGA AAA GGT TAA CTC-3ʹ), 1 µl of template DNA, and ddH_2_O for a final reaction volume of 9 µl. Thermal cycling conditions were: 95 °C for 3 min; 40 cycles of 95 °C for 10 s, 58 °C for 10 s, 72 °C for 30 s; 95 °C for 10 s; and a ramp from 65 °C to 95 °C at a rate of 0.2 °C/10 s for melting curve analysis.

The Cys1534 PCR was based on the protocol described by Yanola et al. [40]. Each reaction contained 4.5 µl of iQ-SYBR Green Supermix (Bio-Rad Laboratories Inc.), 0.45 µl Cys forward primer (5ʹ-GCG GGC AGG GCG GCG GGG GCG GGG CCT CTA CTT TGT GTT CTT CAT CAT GTG-3ʹ), and 0.45 µl each of Phe forward primer (5′-GCG GGC TCT ACT TTG TGT TCT TCA TCA TAT T-3′) and a common reverse primer (5′-TCT GCT CGT TGA AGT TGT CGA T-3′), 1 µl of template DNA and ddH_2_O for a final reaction volume of 9 µl. Thermal cycling conditions were: 95 °C for 3 min; 40 cycles of 95 °C for 10 s, 57 °C for 10 s, 72 °C for 30 s; 95 °C for 10 s; and a ramp from 65 °C to 95 °C at a rate of 0.5 °C/5 s for melting curve analysis.

The Leu410 PCR was performed based on the protocol described by Saavedra-Rodriguez et al. [41]. Each reaction contained 4.5 µl of iQ-SYBR Green Supermix (Bio-Rad Laboratories Inc.), 0.45 µl of each primer (Val forward primer [5ʹGCG GGC AGG GCG GCG GGG GCG GGG CCA TCT TCT TGG GTT CGT TCT ACC GTG-3ʹ], Leu forward primer [5′-GCG GGC ATC TTC TTG GGT TCG TTC TAC CAT T-3′], and a common reverse primer [5′-TTC TTC CTC GGC GGC CTC TT-3′]), 1 µl of template DNA and ddH_2_O for a final reaction volume of 9 µl. Thermal cycling conditions were: 95 °C for 3 min; 40 cycles of 95 °C for 10 s, 60 °C for 10 s, 72 °C for 30 s; 95 °C for 10 s; and a ramp from 65 °C to 95 °C at a rate of 0.2 °C/10 s for melting curve analysis.

### Data analysis

Percentage mortality at the diagnostic time of 30 min was used to describe the susceptibility status of the mosquito populations tested. Populations were classified as resistant and susceptible based on WHO and CDC guidelines [37, 38]: when mortality was < 90%, the population was considered to be resistant; when mortality was ≥ 98%, the population was considered to be susceptible; mortality ranging from 90 to 97% suggested that the population was developing resistance. Percentage mortality was calculated with the 95% CI from the bioassays and for allele frequencies.

Interquartile ranges of the mean of the optical density (OD) values from the biochemical assays were compared between study populations and the susceptible reference strain. Regression analyses were performed to measure the statistical significance of differences between the mean OD values between populations.

Pearson chi-square tests were performed to understand the associations between Gly1016 and Cys1534 genotypes and phenotypes of bioassayed mosquitoes. The population-level allele frequencies were calculated using the following equation [42]:

$$\frac{{n\;{\text{heterozygotes}} + 2\left( {n\;{\text{homozygotes}}} \right)}}{{2\left( {{\text{total}}\;n\;{\text{mosquito}}\;{\text{analyzed}}} \right)}}.$$

The linkage disequilibrium, departures from the Hardy–Weinberg equilibrium (HWE), and the *P *value for Gly1016 and Cys1534 in each population were assessed using Fisher’s exact test in the population genetics software package GENEPOP version 4.2 (https://genepop.curtin.edu.au/) [43]. Statistical analyses were conducted in Microsoft Excel 2016 (Microsoft Inc., Redmond, WA, USA) and Stata 15 (StataCorp LLC, College Station, TX, USA).

## Results

### Insecticide bioassays

In the mosquito populations collected from Dhaka City, *Ae. aegypti* mortality ranged between 0% in Malibagh to 6.7% in Gulshan & Karail at the diagnostic dose of permethrin. A higher dose of permethrin (2× the diagnostic dose) was tested with the populations of Dhanmondi & Mohammadpur and Gulshan & Karail but still resulted in < 50% mortality at the diagnostic time point. In contrast, *Ae. aegypti* mortality at the diagnostic dose of deltamethrin varied between the sampling areas of Dhaka City, ranging from 49.0% (95% CI ± 7.3) in Gulshan & Karail to 100% (95% CI ± 1.6) in Uttara. Susceptibility to malathion was tested in three populations from Dhaka City: the Gulshan & Karail population was resistant (62.9% mortality, 95% CI ± 2.7), and the Dhanmondi & Mohammadpur (98.1% mortality, 95% CI ± 2.7) and Uttara (100% mortality, 95% CI ± 2.1) populations were susceptible. All Dhaka City populations tested against bendiocarb were susceptible (100% mortality in all populations) (Fig. 1).

**Fig. 2:**
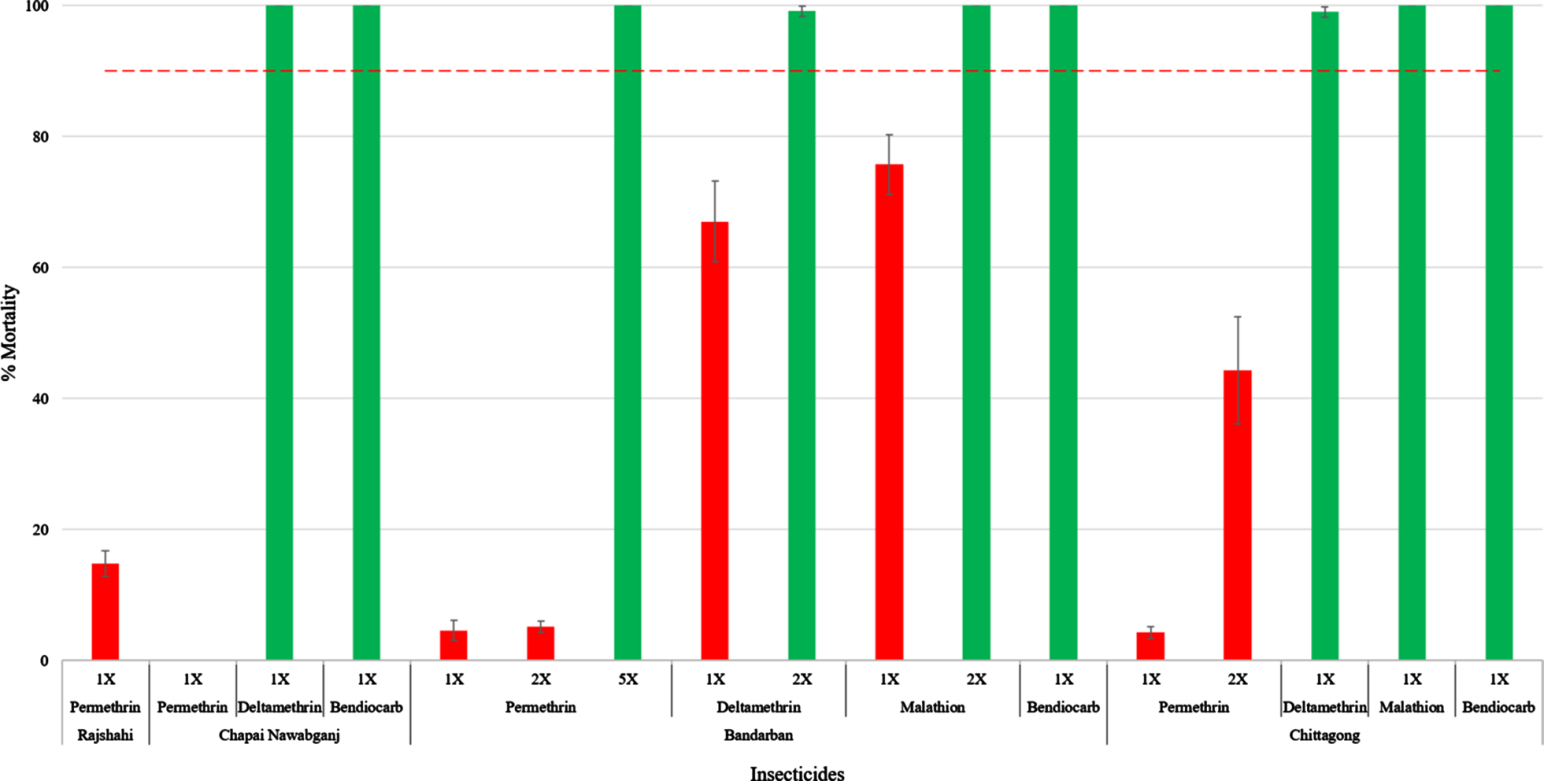
Bioassay results for female *Ae. aegypti* from four non-Dhaka locations. Red bars indicate resistance, green bars indicate susceptibility, and the red dashed line indicates the 90% mortality threshold. Results are given as percentage mortality at 30 min ± 95% CI.* 1X* diagnostic dose,* 2X* twofold diagnostic dose,* 5X* fivefold diagnostic dose

The *Ae. aegypti* populations sampled from the non-Dhaka locations were also highly resistant to permethrin, with mortality ranging from 0% in the Chapai Nawabganj population to 14.8% (95% CI ± 2.0) in the Rajshahi population. When the concentration of permethrin was increased to 2× in Chittagong, mortality was still < 50%. However, when the permethrin concentration was increased to 5× in Bandarban, the population was fully susceptible (100% mortality). While the Chapai Nawabganj (100% mortality, CI ± 0.79) and Chittagong (99.0% mortality, 95% CI ± 0.79) populations were susceptible to deltamethrin, the Bandarban population was resistant to deltamethrin at the diagnostic dose (67% mortality, 95% CI ± 6.1) but susceptible when the concentration was increased to 2× (99.1% mortality, 95% CI ± 0.79). The Bandarban population was also resistant to the diagnostic dose of malathion (75.7% mortality, 95% CI ± 4.6) but susceptible to 2× higher malathion (100% mortality). The ROCK reference strain was confirmed to be fully susceptible to the diagnostic doses of the four insecticides. A summary of bioassay data is presented in Fig. 2.

### Biochemical assays

**Fig. 3:**
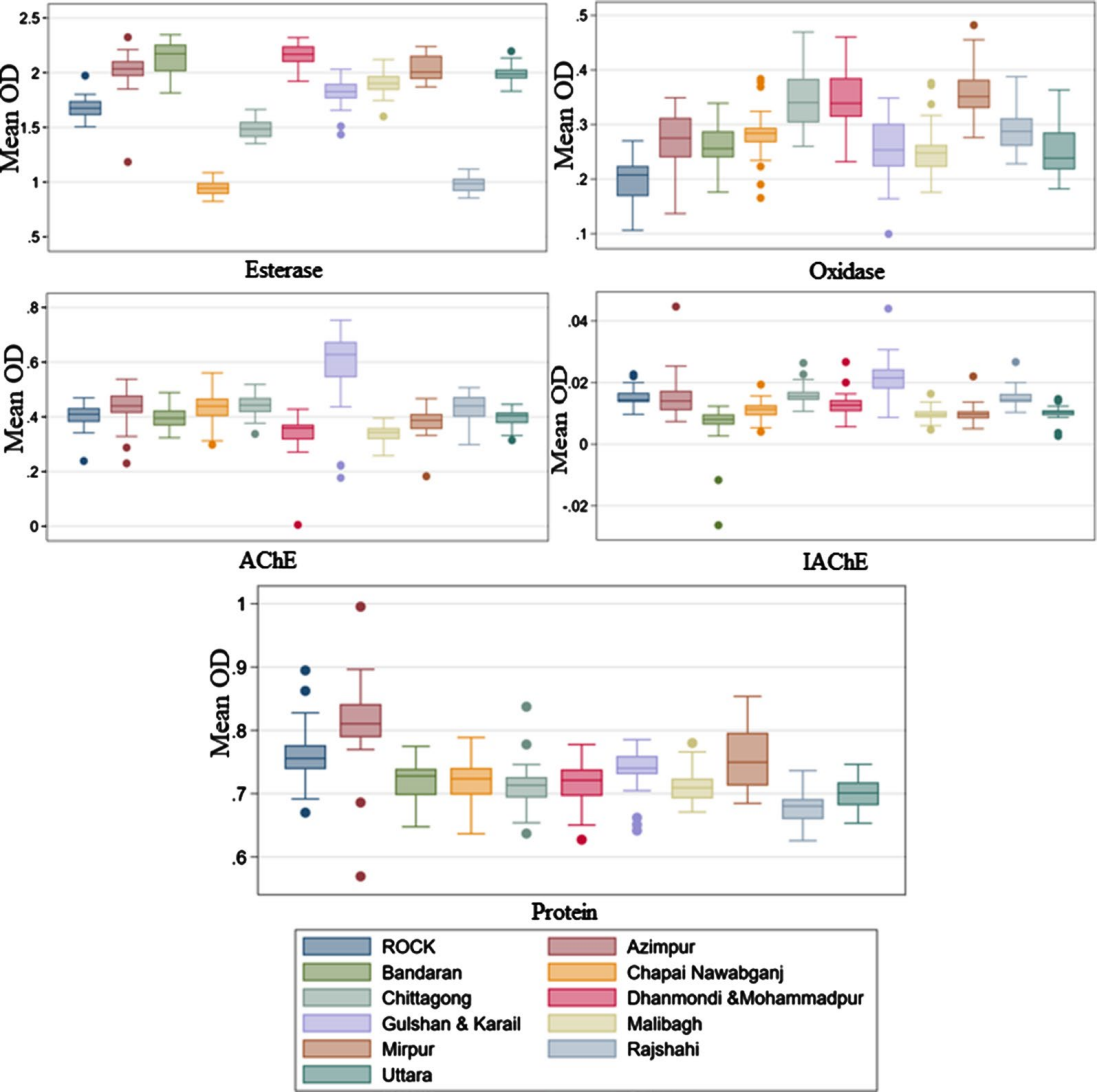
Enzyme activity levels in populations (= different sample areas) of *Ae. aegypti* from Bangladesh compared to the insecticide-susceptible Rockefeller (*ROCK*) *Ae. aegypti* reference strain. Box plots denote the 50th percentile of the mean optical density (*OD*) values, whiskers are the remaining percentile values, and the dots are outliers

All *Ae. aegypti* populations tested from field collections had significantly higher (*P* < 0.0001) MFO levels compared to the insecticide-susceptible ROCK reference strain. β-EST activity levels of *Ae. aegypti* populations from Azimpur, Uttara, Dhanmondi & Mohammadpur, Gulshan & Karail, Malibagh, Mirpur, and Bandarban were significantly (*P* < 0.0001) higher than those of the ROCK reference strain, but β-EST levels in *Ae. aegypti* populations from the non-Dhaka sites of Chapai Nawabganj, Chittagong, and Rajshahi were significantly lower than those of the ROCK strain (*p* < 0.0001). In the case of AChE activity, populations from Azimpur (*P* < 0.042), Chittagong (*P* < 0.019), and Gulshan & Karail (*P* < 0.0001) had significantly higher activity levels than the ROCK reference strain, and those from Dhanmondi & Mohammadpur (*P* < 0.001) and Malibagh (*P* < 0.001) had significantly lower activity levels than the ROCK strain. The estimated levels of IAChE were significantly higher (*P* < 0.0001) in the Gulshan & Karail population compared to the ROCK strain, which suggests that AChE insensitivity may exist in the former population. In comparison, IAChE levels were low across the remaining populations, suggesting that the target site remains sensitive. However, it is noteworthy that levels were significantly lower than those in the ROCK strain in Bandarban, Chapai Nawabganj, Mirpur, and Uttara (*P* < 0.001). When total protein content was compared between mosquito populations, with the exception of Azimpur and Mirpur, total protein content in all populations was significantly (*P* < 0.026) lower than in the ROCK strain, suggesting that body size was generally smaller for most of the field populations (Fig. 3).

### Knockdown resistance (kdr) genotyping

A total of 142 phenotyped mosquitoes from bioassays at diagnostic dose (1×) permethrin and 59 phenotyped mosquitoes from deltamethrin 1X bioassays  were analyzed for the Gly1016 mutation. From the Dhaka mosquito populations exposed to permethrin, 37.8% (28/74) of the survivors (assessed as being alive at the time points) were mutant homozygotes (GG) and 29.7% (22/74) were wild-type homozygotes (VV). The correlations between genotype and phenotype of permethrin-exposed Dhaka mosquitoes were statistically significant (*P* < 0.0001). Most of the dead mosquitoes were wild-type homozygotes (12/14, 85.7%). Among the mosquitoes from sites outside of Dhaka, more than half of the permethrin survivors were heterozygotes (23/44, 52.3%,) and there was an equal number (5/10, 50.0%) of wild-type homozygotes and heterozygotes among the dead mosquitoes. For the deltamethrin bioassays, only dead mosquitoes were genotyped due to limitations at the time of the bioassay. Similar genotype frequencies were seen in the mosquitoes from Dhaka that were dead after exposure to deltamethrin. However, the mosquitoes from outside of Dhaka did not include any mutant homozygotes (Table 2).

**Table 2:** Phenotype and genotype at knockdown resistance (*kdr*) locus 1016 in mosquitoes from Dhaka and non-Dhaka populations exposed to permethrin and deltamethrin

Genotype^a^	Permethrin 1×^b^		Deltamethrin 1×^b^
	Phenotype		Phenotype
	Alive (*n* = 74)	Dead (*n* = 14)	Dead (*n* = 29)
Dhaka			
VV	22 (29.7 %)	12 (85.7%)	9 (31.0%)
VG	24 (32.4%)	1 (7.1%)	9 (31.0%)
GG	28 (37.8%)	1 (7.1%)	11 (37.9%)
*P*	0.000		

Genotype^a^	Permethrin 1×^b^		Deltamethrin 1×^b^
	Phenotype		Phenotype
	Alive (*n* = 44)	Dead (*n* = 10)	Dead (*n* = 30)
Non-Dhaka			
VV	12 (27.3%)	5 (50.0%)	14 (46.7%)
VG	23 (52.3%)	5 (50.0%)	16 (53.3%)
GG	9 (20.5%)	0	0
*P*	0.184		

^a^*GG* Mutant homozygotes, *VV* wild-type homozygotes, *VG* heterozygotes

^b^1× is the diagnostic dose

Of the 170 mosquitoes screened for the Cys1534 mutation, 110 mosquitoes were from permethrin bioassays and the remaining were from deltamethrin bioassays from both Dhaka and non-Dhaka populations. From the permethrin phenotyped Dhaka mosquitoes, 54.1% (33/61) of the resistant (surviving) mosquitoes were 1534 mutant homozygotes (CC) and 41.0% (25/61) were wild-type homozygotes (FF). In the case of permethrin-susceptible mosquitoes, 90.0% (9/10) were FF and the remaining individual was CC. From the non-Dhaka populations, 37.9% (11/29) of permethrin-resistant mosquitoes were CC and 27.6% (8/29) were heterozygotes (FC). Interestingly, none of the permethrin-susceptible mosquitoes from the non-Dhaka sites was FF, and eight of ten were CC. The correlations between genotype and phenotypes of permethrin exposed mosquitoes in both populations were statistically significant (*P* < 0.016 for Dhaka and *P* < 0.043 for non-Dhaka). A total of 60 dead mosquitoes from the deltamethrin bioassays were analyzed for Cys1534. Interestingly, most of the mosquitoes were wild-type homozygotes (FF; 19/30, 65.5%) in Dhaka, whereas the opposite was seen for non-Dhaka populations (Table 3).

**Table 3:** Phenotype and genotype at knockdown resistance *kdr* locus 1534 in mosquitoes from Dhaka and non-Dhaka populations exposed to permethrin and deltamethrin

Genotype^a^	Permethrin 1×^b^		Deltamethrin 1×^b^
	Phenotype		Phenotype
	Alive (*n* = 61)	Dead (*n* = 10)	Dead (*n* = 30)
Dhaka			
FF	25 (41.0%)	9 (90.0%)	19 (63.3%)
FC	3 (4.9%)	0	0
CC	33 (54.1%)	1 (10.0%)	11 (36.7%)
*P*	0.016		

Genotype^a^	Permethrin 1×^b^		Deltamethrin 1×^b^
	Phenotype		Phenotype
	Alive (*n* = 29)	Dead (*n* = 10)	Dead (*n* = 30)
Non-Dhaka			
FF	10 (34.5%)	0	2 (6.7%)
FC	8 (27.6%)	2 (20.0%)	9 (30.0%)
CC	11 (37.9%)	8 (80.0%)	19 (63.3%)
*P*	0.043		

^a^*CC* Mutant homozygotes, *FF* wild-type homozygotes, *FC* heterozygotes

^b^1× is the diagnostic dose

All mosquitoes (*n* = 264) from the permethrin and deltamethrin bioassays (1× and 2×) genotyped for Leu410 were found to be wild-type homozygotes.

**Fig. 4:**
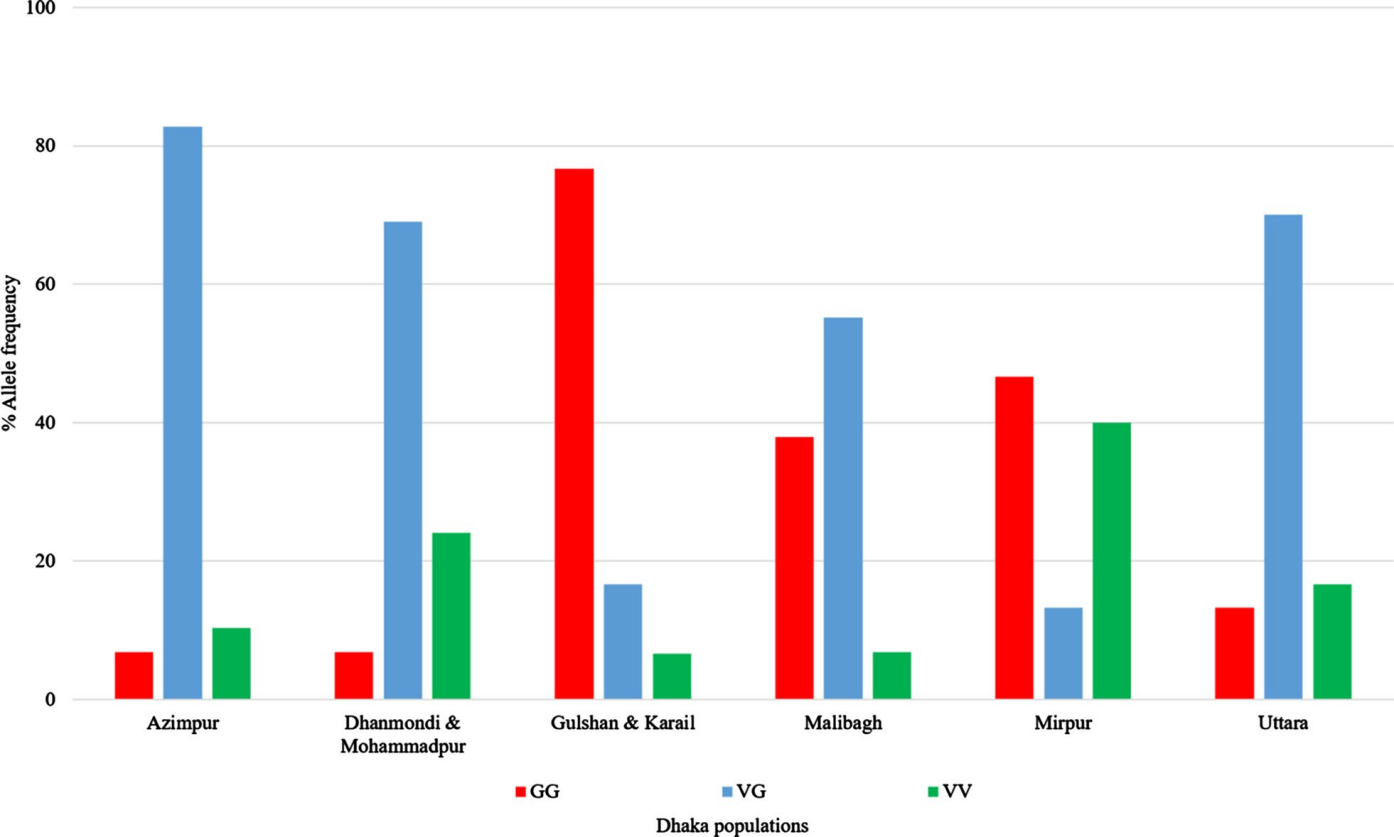
Allele frequencies of the knockdown resistance (*kdr*) mutation Gly1016 in *Ae. aegypti* populations from Dhaka. *GG* Mutant homozygotes, *VV* wild-type homozygotes, *VG* heterozygotes

**Fig. 5:**
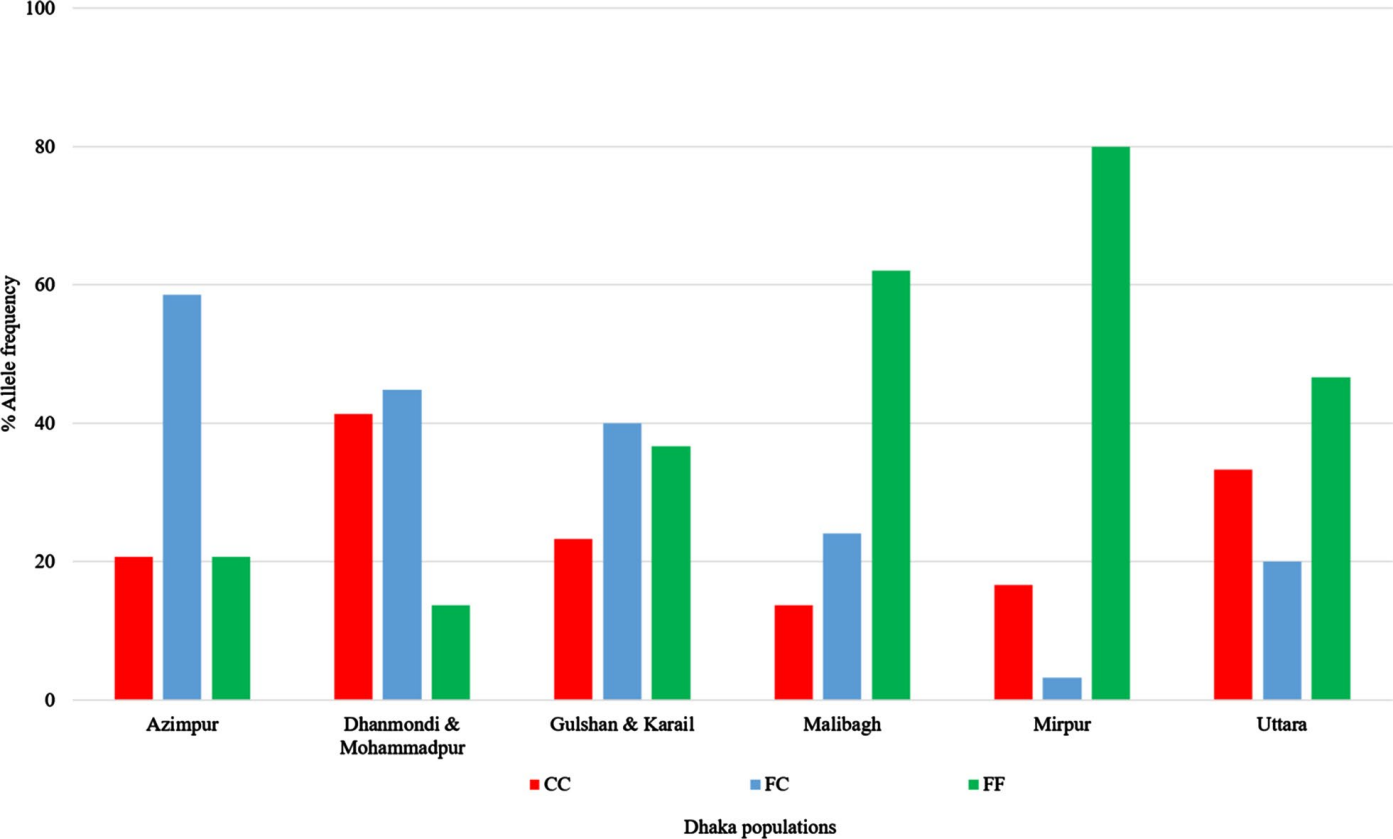
Allele frequencies of the knockdown resistance (*kdr*) mutation Cys1534 in *Ae. aegypti* populations from Dhaka. *CC* Mutant homozygotes, *FF* wild-type homozygotes, *FC* heterozygotes

Of the 177 non-phenotyped mosquitoes from the Dhaka populations, more than half were V1016G heterozygotes (90/177, 51%). The highest Gly1016 homozygote (GG) frequency was observed in the Gulshan & Karail (23/30, 77%) population followed by the Mirpur (14/30, 47%) and Malibagh (11/29, 38%)populations (Fig. 4). In the case of Cys1534, the largest group of the mosquitoes were homozygous wild type (FF) (77/177, 43.5%,). The highest mutant homozygote (CC) frequency was recorded in the Dhanmondi & Mohammadpur population (2/29, 41.4%) (Fig. 5).

The overall allele frequency of Gly1016 and Cys1534 was 57.1% (95% CI ± 8.41) and 38.4% (95% CI ± 5.66), respectively. The Gulshan & Karail population had highest frequency of Gly1016, 85.0% (95% CI ± 30.42), and the Dhanmondi & Mohammadpur population had the highest frequency of Cys1534, 63.8% (95% CI ± 23.22) (Table 4). The HWE test revealed that three populations had significant departures from HWE: the Azimpur population for Gly1016, the Uttara population for Cys1534, and the Mirpur population for both (Table 4).

**Table 4:** Frequency of Gly1016 and Cys1534 *kdr* alleles in *Ae. aegypti* populations from Dhaka

Populations	Allele^a^	*n*	Percentage frequency	95% Confidence interval	*P* value of Harvey–Weinburg equilibrium
Azimpur	G	29	48.3	17.6	0.0008*
	C		50.0	18.2	0.4719
Dhanmondi & Mohammadpur	G	29	41.4	15.1	0.0526
	C		63.8	23.22	1.000
Gulshan & Karail	G	30	85.0	30.4	0.0991
	C		43.3	15.5	0.4540
Malibagh	G	29	65.5	23.9	0.4194
	C		25.9	9.4	0.0546
Mirpur	G	30	55.3	19.1	0.0000*
	C		18.3	6.6	0.0001*
Uttara	G	30	48.3	17.3	0.0654
	C		43.3	15.5	0.0024*

*Significant *P* values

^a^*GG* Gly1016,* CC* Cys1534

**Fig. 6:**
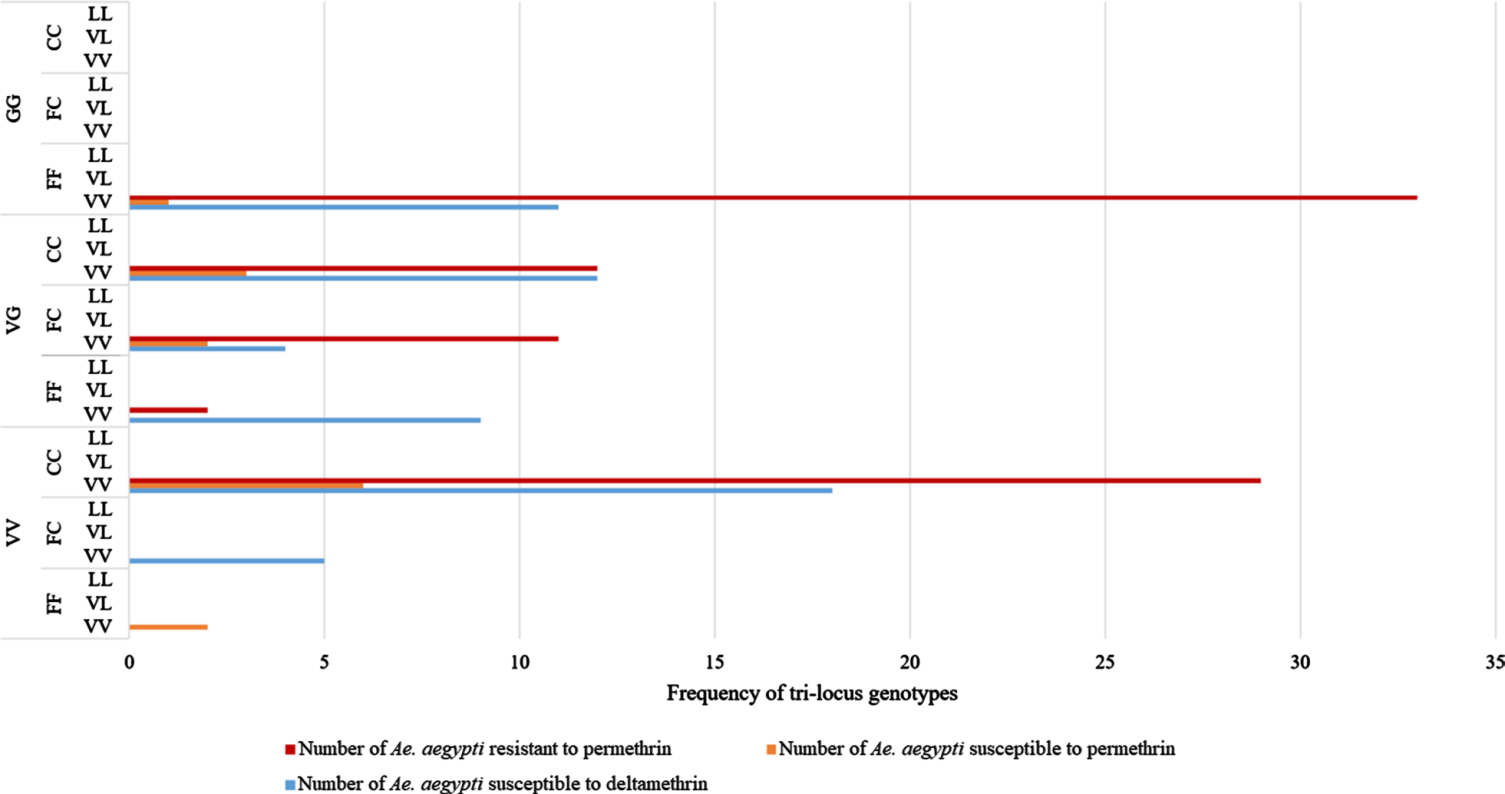
Tri-locus *kdr* genotypes of *Ae. aegypti* from both Dhaka and non-Dhaka sites, by permethrin and deltamethrin resistance phenotypes. The order of the genotypes is V1016G/F1534C/V410L

When all three *kdr* alleles were considered together across the phenotyped mosquitoes, the most common tri-locus genotype was VV/CC/VV (*n* = 53/158) (1016/1534/410) followed by GG/FF/VV (*n* = 45/158). When overall genotype was linked to phenotype, permethrin survivors were most commonly GG/FF/VV (*n* = 33/87). For mosquitoes that were phenotypically susceptible, the most common genotype for those killed by both deltamethrin and permethrin was VV/CC/VV (*n* = 24/73) (Fig. 6).

## Discussion

The application of chemical insecticides either in the form of space sprays, thermal fogging, or LLINs has been carried out for many years in Bangladesh. However, documented evidence of mosquito susceptibility to insecticides is scanty. Some information can be obtained from the ‘Malaria Threat Map’ website on insecticide resistance in some *Anopheles* species [44]. A recent article reported permethrin and deltamethrin resistance in *Anopheles vagus* in the highest malarious region in Bangladesh [34]. However, these reports are limited to phenotypic characteristics, and no clear understanding of resistance mechanisms for any mosquito species is available.

Despite the increasing prevalence of *Aedes*-borne diseases in Bangladesh, the insecticide resistance status of *Ae. aegypti* has previously not been assessed. The results reported here provide a comprehensive overview of insecticide resistance across Dhaka City and in several other sites of high epidemiological importance. We report a high frequency and intensity of permethrin resistance in all mosquito populations that were studied. However, despite this high level of permethrin resistance, susceptibility to deltamethrin was still present in several of the populations. This difference in susceptibility to different pesticides suggests that the underlying mechanisms causing resistant phenotypes in these populations may not be shared across the pyrethroid class of chemicals.

The increased activity of enzymes, including β-ESTs, and increasing levels of MFOs in the populations suggests an important role of metabolic mechanisms in conferring resistance. All of the Dhaka mosquito populations had elevated levels of oxidases and increased esterase activity and they were resistant to permethrin. Outside of Dhaka, esterase activity was notably lower in Chapai Nawabganj and Rajshahi, and while both populations were resistant to permethrin, the latter population remained susceptible to deltamethrin (the former was not tested). Increased activity of esterases and increased levels of oxidases may also be associated with the malathion resistance that was detected previously in the Gulshan & Karail and Bandarban populations [45–48]. In addition, AChE activity was elevated in the Gulshan & Karail population, possibly also contributing to the malathion resistance that was detected there. An important limitation of the biochemical assay data presented in this study is the lack of rigorous normalization of the data based on total protein content. As such, all comparisons were conducted relative to the insecticide-susceptible ROCK control strain. However, the fact that almost all of the field populations had significantly lower total protein content than the susceptible ROCK strain against which they were compared suggests that the increased enzyme levels and activities that were detected could not simply be attributed to greater protein content in the field populations. Additional information on alpha esterases and GSTs could be a focus of future studies. A growing body of evidence suggests that these are important mechanisms in pyrethroid resistance in *Ae. aegypti* [49], with *GSTe2* associated with resistance to both permethrin and deltamethrin, and *GSTe7* associated with resistance to deltamethrin [50, 51].

The *kdr* mutations Gly1016 and Cys1534 were found at varying frequencies in the mosquito populations collected across Dhaka City. This fine-scale spatial heterogeneity suggests that selection pressures for insecticide resistance are variable across small spatial scales within Dhaka City, reflecting trends that have been reported elsewhere [42, 52]. Historically, *Aedes* control in Dhaka and other major cities in Bangladesh solely depends on thermal fogging using a combination of pyrethroid insecticides. Pyrethroids are also commonly used in households via commercially available coils and aerosols. Both operational and domestic insecticide use may contribute to insecticide resistance selection pressures in *Ae. aegypti* [53].

The Gly1016 and Cys1534 *kdr* mutations have been widely reported in mosquito populations in Asia [40, 54, 55]. An additional mutation, Leu410, has also been reported in association with pyrethroid resistance, but its prevalence in Asia has not yet been well studied [23]. Expression of insect sodium channels in *Xenopus* oocytes coupled with electro-neurophysiological measurements has demonstrated that Gly1016, Cys1534, and Leu410 reduce the sensitivity of the VGSC to permethrin and deltamethrin [23, 56]. However, Leu410 was not detected in any of the mosquito populations in the current study. This was an unexpected result, as previous research has suggested the parallel evolution of this mutation together with the polymorphisms at positions 1016 and 1534 [41], both of which were detected at moderate to high frequencies in our study. The co-occurrence of Pro989 with Gly1016 conferring high pyrethroid resistance in *Ae. aegypti* has been reported previously [57]. However, our study did not any analysis of the S989P *kdr* mutation.

In Dhaka City, 1016G and 1534C homozygous mutants were mostly associated with survival in the permethrin bioassays. It is also worth noting that the population from the Dhaka City neighborhood of Mirpur was resistant to permethrin yet susceptible to deltamethrin and was also the population with the highest frequency of Val1016 and Phe1534 wild-type homozygotes. These findings suggest that while *kdr* alleles may be contributing to the insecticide resistance that was detected, they are not the only mechanism and such relationship is not rare [57, 58].

From an operational perspective, the data presented here will be important in guiding the choice of vector control tools. Given the widespread and intense permethrin resistance that was detected, vector control products containing alternative compounds should be used. Although some populations have remained susceptible to deltamethrin, given the high degree of permethrin resistance, it would be prudent to search for alternatives outside of the pyrethroid class. Particularly notable was the detection of deltamethrin resistance in mosquitoes collected in the Bandarban region, where deltamethrin-treated bed nets are routinely used for malaria control [34]. Bandarban was also the only non-Dhaka site to show significantly elevated esterase activity, suggesting that the population was experiencing comparatively greater selective pressure across multiple mechanisms as compared to the other non-Dhaka sites. Vector control activities have focused largely on malaria vectors and have not routinely targeted *Aedes* in this part of Bangladesh. The finding that the *Aedes* population was resistant to the insecticide relied upon for malaria control highlights the importance of implementing strategies based on integrated vector management in Bandarban.

The only insecticide to which every population tested was susceptible was bendiocarb. However, there is no product registered in Bangladesh that could be employed for *Aedes* control that contains bendiocarb as an active ingredient. The next best candidate was malathion, with public health agencies desperately seeking alternatives to pyrethroids. Nevertheless, malathion resistance was detected in several of the populations studied, both inside and outside of Dhaka city. Also, malathion has been used in agriculture for many years in Bangladesh, so selection pressure outside of vector control already exists to a certain degree [59, 60]. In such a scenario as we detected in Bangladesh with a patchwork of insecticide-resistant phenotypes, it will be challenging to find a ‘one size fits all’ solution for *Aedes* control.

## Conclusions

This current study provides evidence of insecticide resistance in *Ae. aegypti* and data on resistance mechanisms, including detoxification enzymes and *kdr* mutations, in Bangladesh. High pyrethroid resistance may be compromising the existing *Aedes* control strategies, and the presence of multiple resistance mechanisms poses further challenges regarding alternatives. Continuous surveillance of insecticide resistance will enable trends in susceptibility to be monitored over space and time and will provide a more robust evidence base upon which to select the most effective vector control tools and strategies. In cities like Dhaka, where operational control faces challenges posed by insecticide resistance, in addition to the rational use of chemicals, sustainable and alternative tools like biocontrol approaches should be considered.

## Impact

The preliminary results were disseminated among different stakeholders and mosquito control authorities immediately after the data were analyzed. Followed by the outbreak of dengue during the monsoon season of 2019, these research findings and recommendations were reinvestigated by the policymakers. As a result, permethrin has been replaced by malathion for the control of adult mosquitoes in Dhaka city [61, 62].

## Supplementary information

**Additional file 1.** All enzyme data.

**Additional file 2.** All phenotype and genotype data.

## Data Availability

All data generated or analyzed during this study are included in this published article as Additional files 1 and 2.

## Abbreviations

AChE: Acetylcholine esterase
BI: Breteau index
β-EST: β-Esterase
DDT: Dichlorodiphenyltrichloroethane
DTNB: Dithio-bis-2-nitrobenzoic acid
GSTs: Glutathione *S*-transferases
HWE: Hardy–Weinberg equilibrium
IRS: Indoor residual spraying
IAChE: Insensitive acetylcholine esterase
icddr,b: International Centre for Diarrhoeal Disease Research, Bangladesh
kdr: Knockdown resistance
LLINs: Long-lasting insecticidal nets
MFOs: Mixed-function oxidases
ROCK: Rockefeller
CDC: U.S. Centers for Disease Control and Prevention
VGSC: Voltage-gated sodium channel
